# Silver nanoparticle protein corona and toxicity: a mini-review

**DOI:** 10.1186/s12951-015-0114-4

**Published:** 2015-09-04

**Authors:** Nelson Durán, Camila P. Silveira, Marcela Durán, Diego Stéfani T. Martinez

**Affiliations:** Biological Chemistry Laboratory, Institute of Chemistry, University of Campinas, CP 6154, Campinas, SP CEP 13083-970 Brazil; NanoBioss Laboratory, Institute of Chemistry, University of Campinas, Campinas, SP Brazil; Brazilian Nanotechnology National Laboratory (LNNano), Brazilian Center for Research in Energy and Materials (CNPEM), Campinas, SP Brazil; Urogenital Carcinogenesis: Urogenital and Immunotherapy Laboratory, Institute of Biology, University of Campinas, Campinas, SP Brazil

**Keywords:** Silver nanoparticles, Protein corona, Cytotoxicity

## Abstract

Silver nanoparticles are one of the most important materials in the nanotechnology industry. Additionally, the protein corona is emerging as a key entity at the nanobiointerface; thus, a comprehensive understanding of the interactions between proteins and silver nanoparticles is imperative. Therefore, literature reporting studies involving both single molecule protein coronas (i.e., bovine and human serum albumin, tubulin, ubiquitin and hyaluronic-binding protein) and complex protein coronas (i.e., fetal bovine serum and yeast extract proteins) were selected to demonstrate the effects of protein coronas on silver nanoparticle cytotoxicity and antimicrobial activity. There is evidence that distinct and differential protein components may yield a “protein corona signature” that is related to the size and/or surface curvature of the silver nanoparticles. Therefore, the formation of silver nanoparticle protein coronas together with the biological response to these coronas (i.e., oxidative stress, inflammation and cytotoxicity) as well as other cellular biophysicochemical mechanisms (i.e., endocytosis, biotransformation and biodistribution) will be important for nanomedicine and nanotoxicology. Researchers may benefit from the information contained herein to improve biotechnological applications of silver nanoparticles and to address related safety concerns. In summary, the main aim of this mini-review is to highlight the relationship between the formation of silver nanoparticle protein coronas and toxicity.

## Background

Recently, silver nanoparticles have received a great deal of attention because of their distinctive physicochemical and biological properties. This interest originated with the use of silver nanoparticles in many different products because of their exceptionally small size and their potential antibacterial effect [1–3]. Currently, silver nanoparticles are primarily used for catalysis, transport, sensing and many other biological and medical applications [4, 5]. However, these applications have increased the possibility of living organisms being directly or indirectly exposed to silver nanoparticles, which could induce numerous deleterious effects on human health and the environment. The adverse effects (primary or secondary) of silver nanoparticles on organs can extend into the cardiovascular or central nervous system, thereby causing neurotoxicity or immunotoxicity [6, 7].

## Important aspects of interactions between silver nanoparticle and cellular systems

**Fig. 1:**
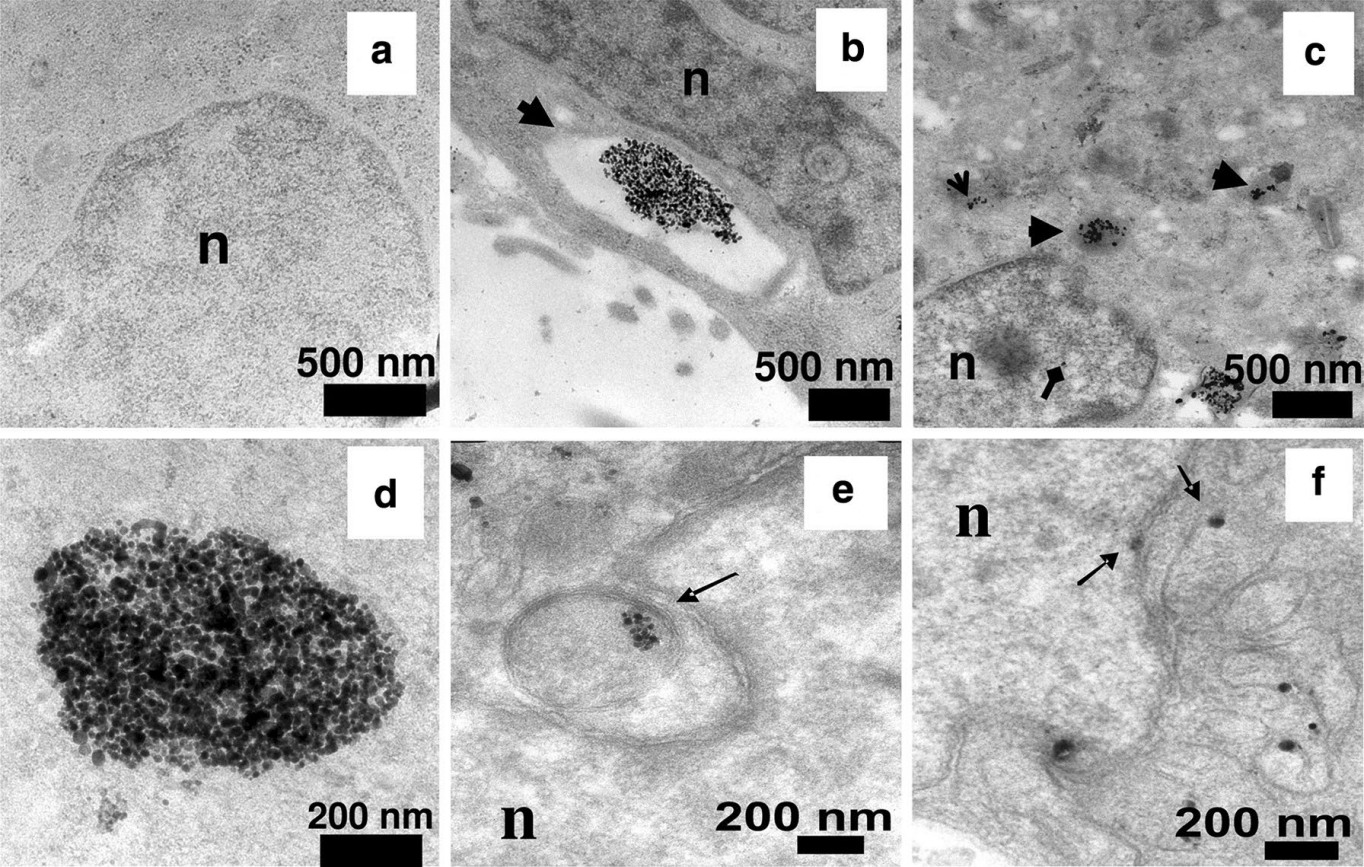
TEM images of ultrathin sections of cells. Untreated cells showed no abnormalities (**a**), whereas cells treated with silver nanoparticles showed large endosomes near the cell membrane with many nanoparticles inside (**b**). Electron micrographs showing lysosomes with nanoparticles inside (*thick arrows*) and scattered in cytoplasm (*open arrow*). *Diamond arrow* shows the presence of the nanoparticle in the nucleus (**c**). Magnified images of nanogroups showed that the cluster is composed of individual nanoparticles rather than clumps (**d**). Image shows endosomes in cytosol that are lodged in the nuclear membrane invaginations (**e**) and the presence of nanoparticles in mitochondria and on the nuclear membrane (**f**) (reproduced from ref. Asharani et al. [14]. by permission of American Chemical Society)

The cytotoxicity and genotoxicity of silver nanoparticles depends on many factors (e.g., concentration, dispersion, size and surface functionalization) [8–11]. For example, reports have indicated that the size of silver nanoparticles is an important factor for cytotoxicity and genotoxicity, probably acting through apoptosis and necrosis mechanisms [12, 13]. Exposure of the human body to silver nanoparticles can occur through different routes (e.g., inhalation, ingestion, injection or physical contact with cuts or wounds); caution is necessary because some in vitro data suggest that even low concentrations of silver nanoparticles can be toxic in some cases [14–17]. Asharani et al. [15] showed by transmission electron microscopy (TEM) that silver nanoparticles can penetrate into cellular compartments such as endosomes, lysosomes and mitochondria (Fig. 1).

**Fig. 2:**
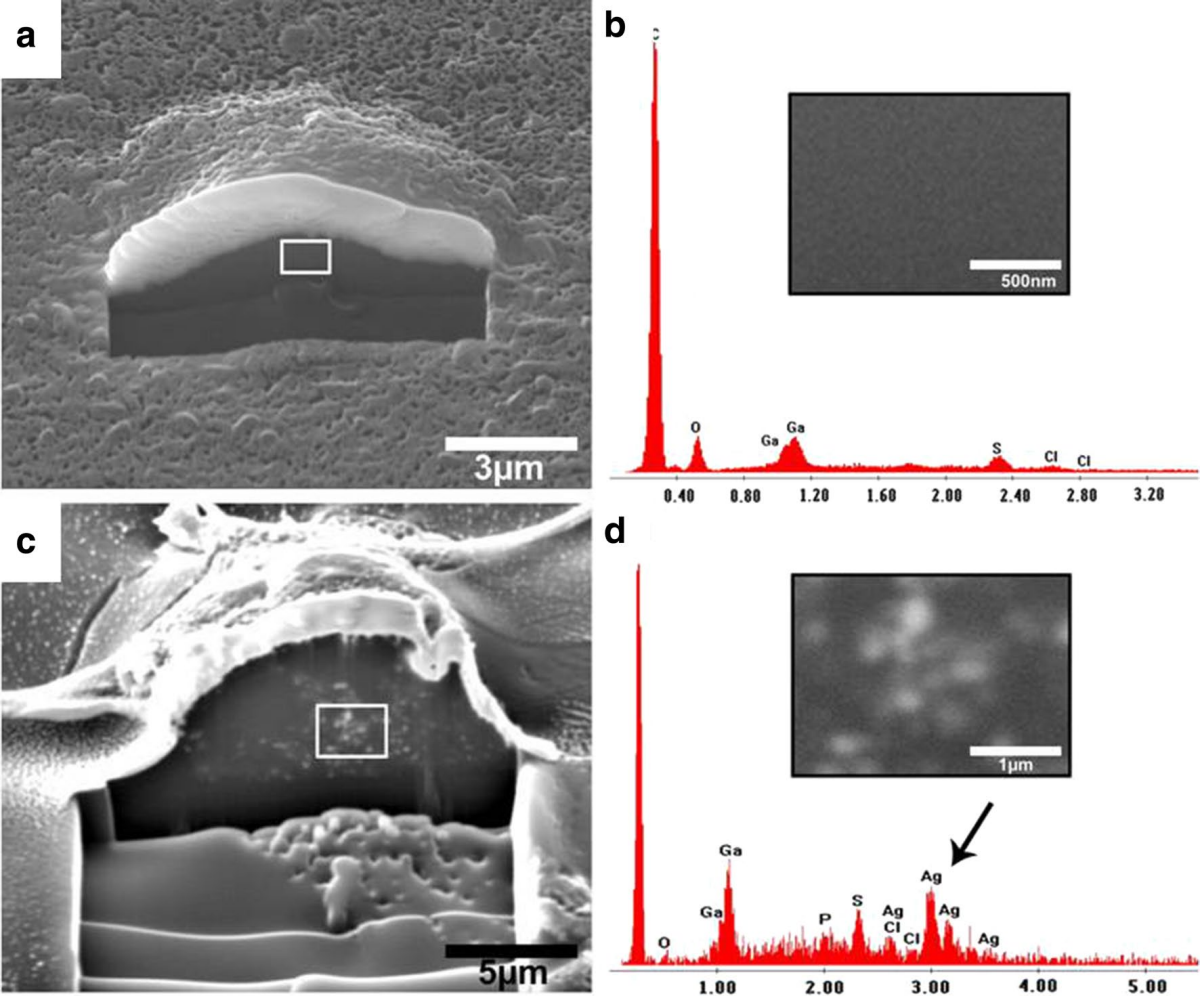
Detection of silver nanoparticles after an incubation time of 24 h inside hMSC by FIB/SEM (**a**, **c**) and the corresponding elemental analysis (**b**, **d**). The cells were cultured for 24 h with 50 µg ml^−1^ silver nanoparticles (**c**, **d**) or without Ag-NP (**a**, **b**). A part of the gold-sputtered hMSC and the surface was cut by ion milling in order to visualize the internalized particles. The EDX spectra (**b**, **d**) show the detected elements; the *black arrow* denotes silver within the milled cell (**d**). The insets in **b** and **d** represent the enlarged area denoted by the white frames in Fig. 1a, c (reproduced from ref. Greulich et al. [9], by permission of Elsevier Ltd.)

Many types of cells that interact with silver nanoparticles have been cultured and studied, including red blood cells, BRL3A rat liver cells, PC-12 neuroendocrine cells, GSCs germ line stem cells, RBE4 rat brain endothelial cells, MCF-7 human breast adenocarcinoma cells, HepG2 human liver cells, BEAS-2B bronchial epithelial cells, A4549 lung alveolar epithelial cells, and hMSC human mesenchymal stem cells (Fig. 2) [9, 18–30]. Thus far, data collected in vitro and in vivo indicate that the production of reactive oxygen species (ROS) plays an important role in the toxic effects of silver nanoparticles [31, 32] and is responsible for many changes (e.g., molecular and biochemical) related to genotoxicity in cultured cells (e.g., DNA breakage) [33]. It is also stated in the literature that the dissolution of silver nanoparticles may have a key role in their toxicity [34, 35]. Moreover, many studies have suggested that the antimicrobial activity of silver nanoparticles on different types of pathogens depends on oxidative stress [36–39].

**Fig. 3:**
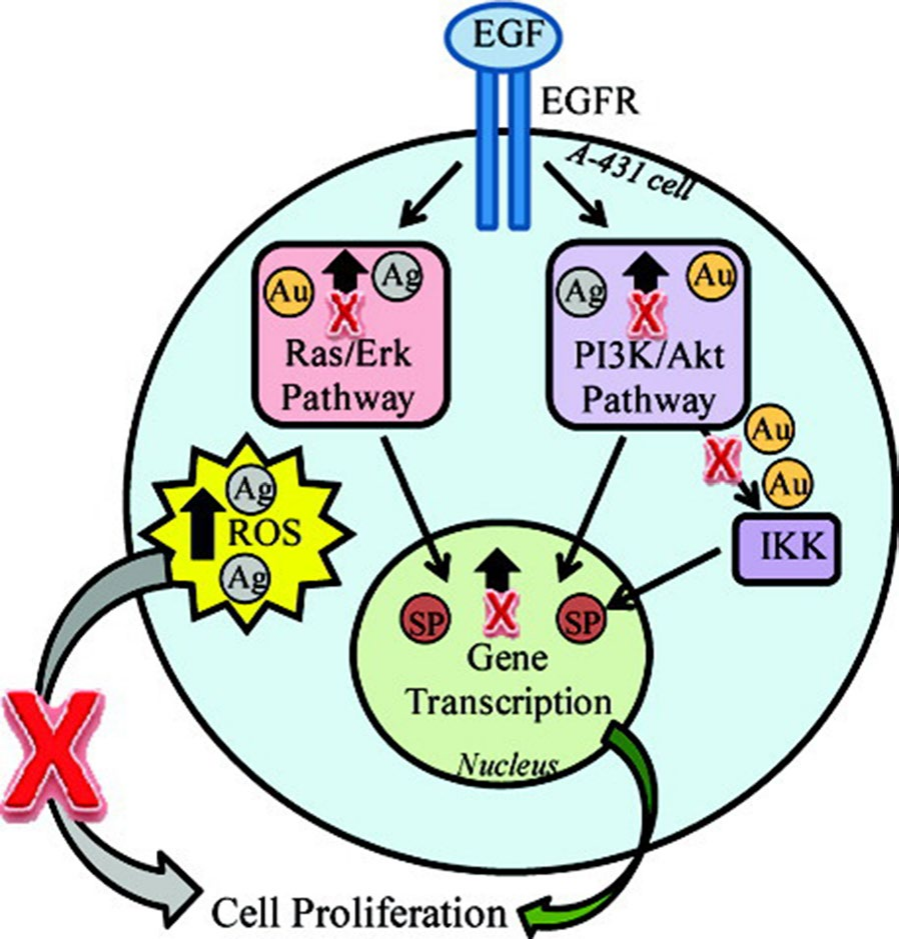
Sites of cellular disruption by metallic nanoparticles. This model depicts the different cellular events in which silver (Ag), and gold (Au) nanoparticles were found to interfere (reproduced from ref. Comfort et al. [34] by permission of American Chemical Society)

Knowledge of the chronic toxic effects that result from low-level exposure to silver nanoparticles is limited. A study of 10 nm silver, iron and gold nanoparticles demonstrated that all particles impeded epidermal growth factor (EGF)-dependent signal transduction, but by different mechanisms, as shown in Fig. 3. Silver nanoparticles produced a high ROS level and diminished serine/threonine protein kinase (Akt) and small guanosine triphosphate-binding protein/extracellular signal-regulated kinase (Erk) signaling. Silver nanoparticles significantly diminished the phosphorylation of Akt and Erk and inhibited Akt activity. Comfort et al. [36] stated that pretreatment with these metallic nanoparticles drastically interfered with the cellular response to EGF. Moreover, they reported that the major challenge is to be able to correlate the data obtained using an in vitro model and extrapolate the results to an in vivo system.

In an industrial environment, the most relevant occupational health risk from exposure to silver nanoparticles is inhalational; therefore, an important study was carried out with the A549 cell line, which is widely used as an in vitro model of pulmonary epithelial cells for nanotoxicity studies [40]. The authors found that the toxicity of silver nanoparticles to these cells were mediated via ROS-dependent and also ROS-independent pathways [40].

When investigating the biological activities of silver nanoparticles, it is important to recognize that the nanoparticles will always interact with a protein medium, such as cell culture medium or a bacterial culture, prior to their biological actions. Therefore, the presence of a protein corona could make a vast difference to their biological activity. Consequently, it is important to review these topics and how they relate specifically to the interactions of silver nanoparticles with biological systems because this nanomaterial is exceedingly important in the market [6, 41].

## General aspects of protein corona

**Fig. 4:**
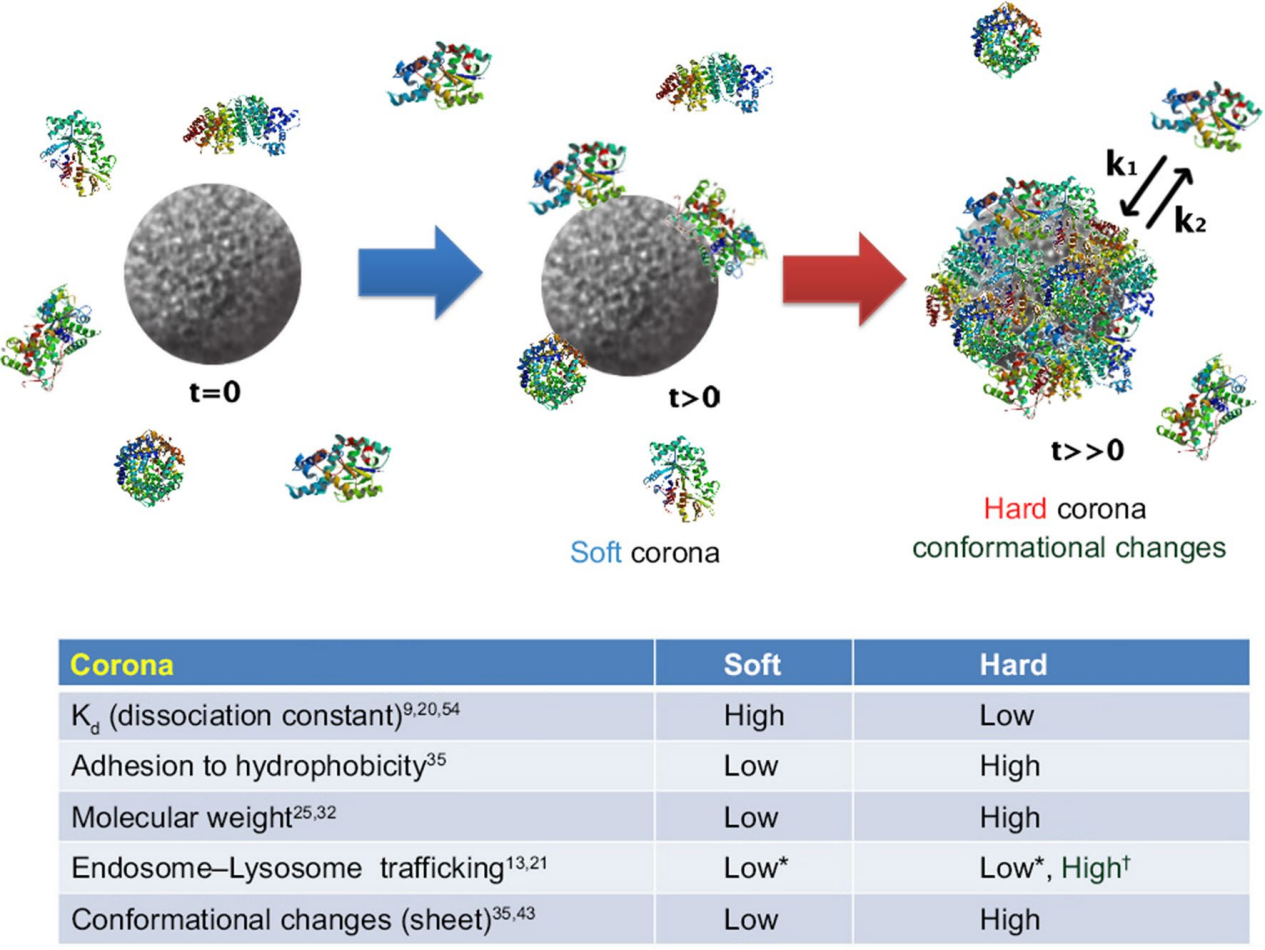
Schematic illustration and characteristics of a hard and a soft corona. The protein corona encompassing the nanoparticles. Hard coronas are characterized by slow exchange (i.e., several hours) and lower abundance, with a high affinity of proteins, whereas soft coronas are typified by rapid exchange (i.e., several minutes) and lower affinity of proteins with weakly bound outer layers on nanoparticles. There is a different response of cellular and biochemical factors by soft and hard corona formation. *Compared with serum-free condition. ^†^Compared with soft corona (modified from ref. Lee et al. [41])

The corona formation process depends on competition between proteins to be adsorbed onto the surface of nanoparticles. Miclaus et al. [42] suggested that the nanoparticle-protein interaction is dynamic, with exchanges between surface-bound and bulk proteins occurring on different time scales. Proteins with higher affinity for the surface exchange slowly, forming the hard corona, the innermost layer composed of tightly bound proteins (over longer periods of hours). Other proteins with low affinity are very quickly replaced, forming the soft corona, the outer layer composed of loosely bound proteins with lower affinity for the nanoparticle surface (over short time scales of seconds or minutes) (Fig. 4).

To better understand the interactions between nanoparticles and biological fluids, many studies have been performed in vitro and/or in vivo on tissues, on cells [42] and especially on proteins [44, 45]. All current data indicate that it is the proteins contained in the corona and not the nanoparticles themselves that interact with the cells [46, 47]. Therefore, the nanoparticle-corona complex is a new entity that represents “what the cell sees”, with the corona being the interface between the nanoparticle and the cellular system [46]. Thus, under physiological conditions, the protein corona that forms on the surfaces of nanoparticles determines their biological identities [48–54]. It has been suggested that this protein corona is more significant in determining the biological response (i.e., immunogenicity) than the bare material properties of the particle itself [43].

The parameters that affect corona composition include particle size [50], particle shape [55] and particle surface [56] properties as well as biological fluid properties and composition [47]. The formation of both soft and hard coronas is an equilibrium process in which the protein composition is directed by the initial concentration of each protein in the medium as well as its off rate (k_back_) [42]. Corona formation and composition have important implications for both toxicity [43] and internalization [57].

Many studies have been performed on the long-lived, hard corona because it can be separated from the incubation medium through subsequent centrifugation and washing steps. However, it is important to note that this corona represents the proteins with longer residence times than the period of the washing steps in the separation process [58]. Recently, Del Pino et al. [59] determined that each washing step causes a decrease in the bulk biomolecule concentration, which shifts the equilibrium of bound and unbound proteins and specifically removes the faster exchanging fraction [42].

These aspects are all related, in general, to different types of nanoparticles. Therefore, the overview herein will discuss a more specific topic, silver nanoparticles, and what has been published in this important area once silver is the most widely used nanostructured material with many potential effects in biological systems and even on environmental behavior [2, 4, 6, 60–65].

## Specific aspects of silver nanoparticle protein coronas

### Proteins

#### Bovine serum albumin (BSA)

One of the first papers on protein coronas [51] used the structure of the bovine serum albumin (BSA) molecule because BSA is an adequate model system for the study of nanoparticle/protein interactions. In this study, two chemically synthesized silver nanoparticles with either a 20 nm diameter (3 × 10^14^ nm^2^ mL^−1^ surface area per mL) or a 40 nm diameter (3 × 10^15^ nm^2^ mL^−1^) capped with citrate and 70 nm silver nanoparticles capped with polyvinylpyrrolidone (PVP) were incubated with BSA. The α-helix content of the protein was analyzed using circular dichroism spectroscopy, which was based on different spectral intensities at 208 and 220 nm. BSA primarily contains α-helices as its secondary structural elements, and the loss of the structure of this protein shows the degree of disruption of interaction between BSA and the nanoparticles. The BSA signal decreased with an increase in the surface area of citrate-coated silver nanoparticle present in the mixture. The authors showed that the nanoparticle surface area required to remove free protein from the bulk solution corresponded well to the calculated amount of protein that could be adsorbed in a monolayer on the surface of the nanoparticles. Compared to citrate-stabilized silver nanoparticles, the interaction of BSA with PVP-stabilized silver nanoparticles revealed a different fate. Although the signal behavior showed similar aspects to the spectra run for citrate-coated silver nanoparticles, a completely different behavior was observed as the surface area of the nanoparticles was increased. The band at 208 nm decreased significantly compared to the second relative minimum at approximately 220 nm, which was completely different from citrate-silver nanoparticles and probably means that the mechanistic interpretation is more complex than in the case of citrate.

Treuel et al. [66] demonstrated that the behavior of proteins (such as BSA) towards nanoparticles could be strongly affected by the presence of polymer coatings, as previously discussed. Surface-enhanced Raman spectroscopy was used for further elucidation of protein binding to silver surfaces. The first interpretation of the differences in binding affinities for the citrate silver nanoparticles and the PVP silver nanoparticles was that the strong interactions between sulfur-containing groups in the protein and the metal surface was prevented when a polymer coating was present. The authors suggested that nanotoxicology might benefit from this information, which could be used to improve nanomedicinal applications. By using a wide range of polymer surface coatings, the biological transport and medical action of nanoparticles could be optimized. In the case of multiple-protein coronas, simulating real in vivo situations, the competition between the proteins present the medium is very expressive, thus the nanoparticle surface properties become even more relevant.

**Fig. 5:**
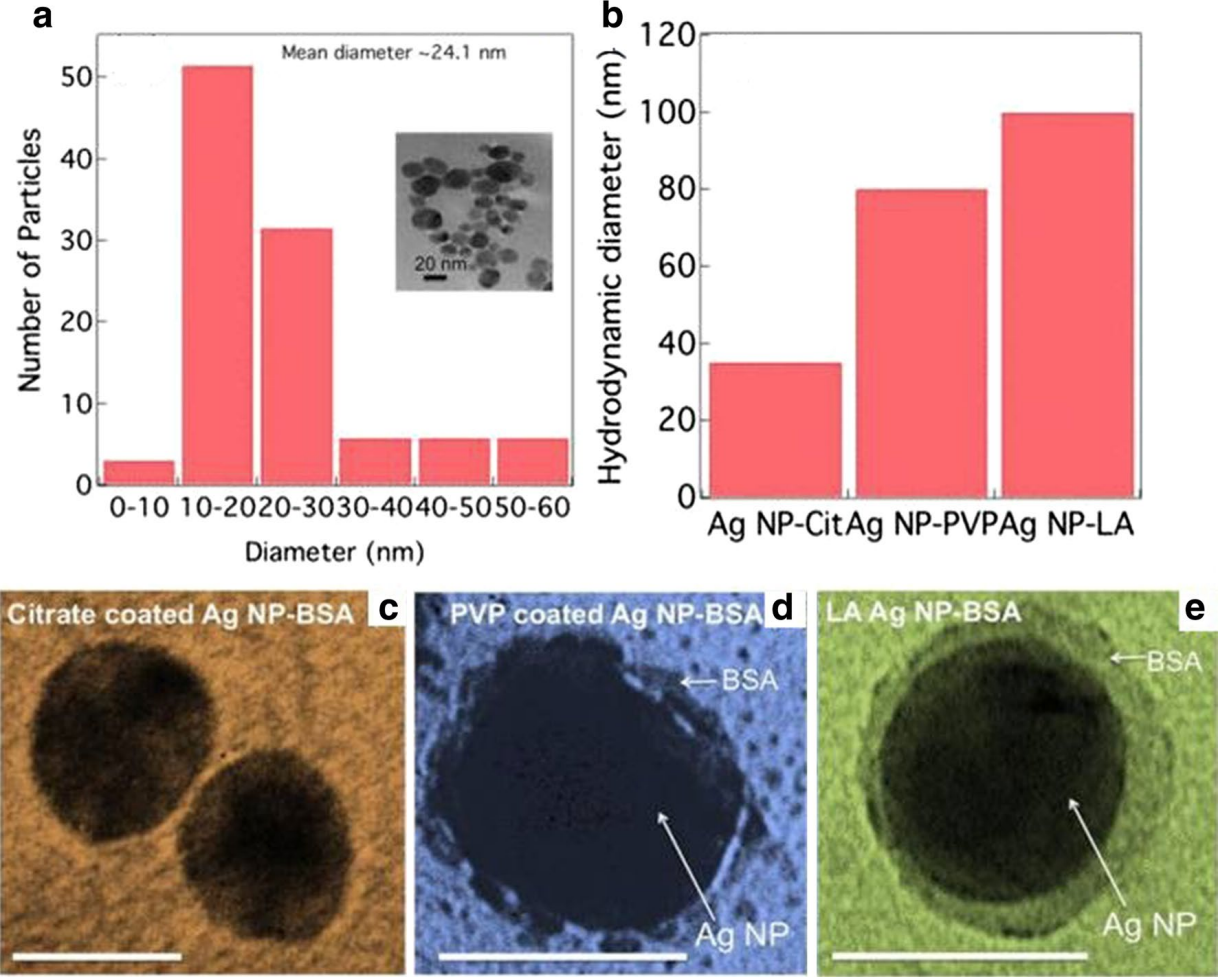
**a** The diameter distribution for laser ablated silver nanoparticles (La-silver nanoparticles) exhibits a unimodal distribution with an average 24 nm. The *inset* shows a representative TEM image for La silver nanoparticles. **b** The hydrodynamic diameter for BSA coated silver nanoparticles (BSA-silver nanoparticles) is different for different surface coatings. TEM images for BSA coated citrate coated silver nanoparticles (Citrate-silver nanoparticles) (**c**) and PVP coated silver nanoparticles (PVP-silver nanoparticles) (**d**) and La-silver nanoparticles (**e**) show that the coating is maximum for La silver nanoparticles in agreement with the hydrodynamic diameter. The *scale bar* for all the panels is 20 nm. (reproduced from ref. Podila et al. [67], by permission of American Institute of Physics)

Similar results were published with different methodologies regarding albumin-silver nanoparticle interactions [67, 68]. Podila et al. [69] investigated the physiological and chemical interactions of a simple protein corona by studying the combination of a specific protein, such as BSA, with PVP- and citrate- stabilized silver nanoparticles. TEM images (Fig. 5a) confirmed that the uncoated silver nanoparticles produced via laser ablation (La-silver nanoparticles) were spherical with an unimodal diameter distribution (24 nm). The average diameter of citrate- and PVP-coated silver nanoparticles was approximately 20 nm. In the investigation of protein binding, an adequate amount of BSA (0–15 nM) was added to the silver nanoparticle suspension (10 µM) and thoroughly mixed via stirring to achieve good dispersion. Then, the suspensions were incubated at 37 °C for 1 h.

It can be observed in Fig. 5c–e that the La-silver nanoparticles exhibited more protein coating than the citrate- or PVP-coated silver nanoparticles. Moreover, the citrate- and PVP-coated silver nanoparticles aggregated upon exposure to proteins, indicating that their electrostatic stabilization groups were displaced from their surfaces.

In agreement with the TEM results (Fig. 5a), the dynamic light scattering results indicated that the Laser ablation (La)-silver nanoparticles supported the highest saturation amount of BSA compared to the citrate-and PVP-coated silver nanoparticles (Fig. 5b).

The ratio of the areas under the β-sheets to α-helices (I_1630_/I_1650_) by FTIR provided a quantitative measure for the net change in protein entropy. Native BSA had a ratio of <1, indicating an ordered form (higher α-helices). After the interaction of BSA with the nanoparticles, the ratios were greater than 1 (citrate-silver nanoparticles ~1.5, PVP-silver nanoparticles ~2.5 and La-silver nanoparticles ~9.5), indicating that all nanoparticle types induced an increase in the protein conformational entropy, or a decrease in α-helix content. Additionally, the change in conformational entropy was highest for the uncoated La- silver nanoparticles, consistent with previously observed protein adsorption on bulk surfaces. Finally, the authors found that uncoated and surfactant-free silver nanoparticles prepared using a physical process produced maximal BSA coatings owing to their increased changes in entropy. However, BSA exhibited a relatively lower affinity for electrostatically stabilized nanoparticles because it exerted constrained entropy changes.

This finding was similar that of Shannahan et al. [70], who found that 20 nm silver nanoparticles bound more strongly to hydrophobic proteins than did 110 nm silver nanoparticles. These authors suggested that the complexity and abundance of the protein corona constituents, in general, were related to the free energy in protein folding-unfolding induced by different surface components for silver nanoparticles of the same size. However, the differences in protein corona formation for 20 and 110 nm silver nanoparticles were most likely due to their surface curvature and the energy involved in protein adsorption and agglomeration. In other words, this report demonstrated the importance of electrostatic and hydrophobic interactions in protein corona formation, showing the broad biological and toxicological implications of these interactions. It is possible to conclude that the surface stabilization by different capped materials, a different BSA interaction occurred. The comparison of bare nanoparticles (laser ablation) versus citrate- or PVP-coated nanoparticles showed coated silver nanoparticles aggregated upon exposure to BSA, indicating that their electrostatic stabilization groups were offset from their surfaces. Then, BSA exhibited a relatively lower affinity for electrostatically stabilized nanoparticles because it exerted constrained entropy changes, thus, demonstrating the importance of electrostatic and hydrophobic interactions in protein corona formation from BSA.

#### Fetal calf and bovine serum

To understand PVP-stabilized silver nanoparticles, several protein moieties were studied. PVP-stabilized silver nanoparticles agglomerated and precipitated very fast in protein-free RPMI cell culture medium, which contains serum proteins [71, 72]. BSA partially prevented this agglomeration, but fetal calf serum (FCS) prevented the agglomeration entirely. The interaction of these two capped silver nanoparticles with FCS showed slight toxic effects against human mesenchymal stem cells. The toxicity of the silver nanoparticles is probably due to the release of silver ions and the BSA and FCS probably bind to these ions, thus avoiding their toxicity, although other factors can be acting simultaneously. It is also known that BSA and silver nanoparticles form complexes by van der Waals and electrostatic forces, and these metal ion–albumin complexes are easily internalized by cells [73]. It is worth to notice that the formation of the corona around the silver nanoparticles may also affect their dissolution rates, likely affecting their overall toxicity. Therefore, the true nature of the protective effect of proteins needs further study, and silver nanoparticles must be used with care because their agglomeration behavior and the presence of proteins play important functional roles [71, 72].

**Scheme 1:**
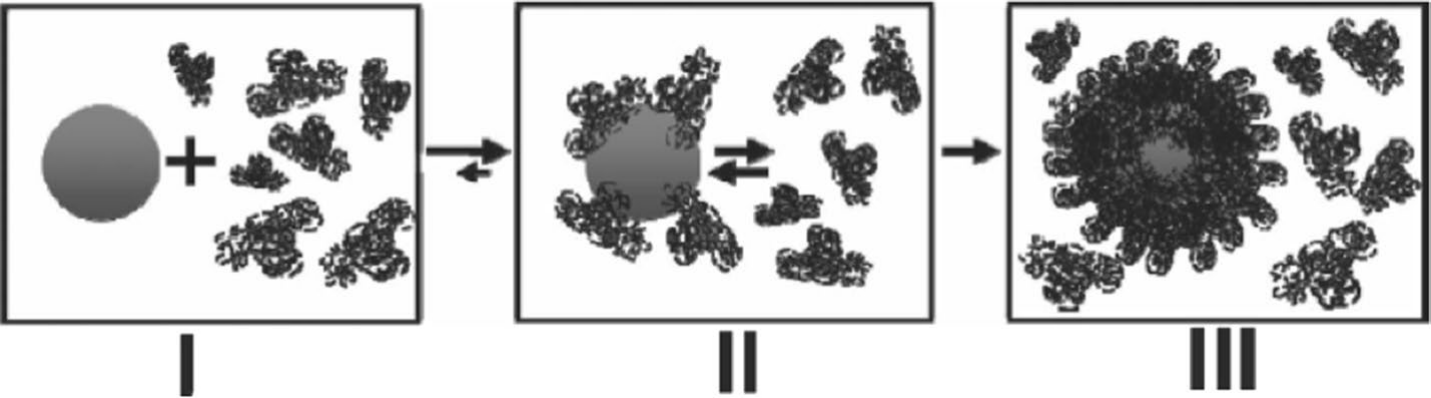
NP–protein interactions. The process of conjugation of the NP when inserted in biological media takes few minutes in the working conditions (**I**), which evolves to a NP coated with protein in equilibrium with the proteins in the medium (**II**), then later evolves towards an irreversible protein corona with proteins that are no longer in equilibrium with their in-solution counterparts (**III**) (reproduced from ref. Casals et al. [72] by permission of Wiley–VCH Verlag GmbH & Co. KGaA, Weinheim)

To study the bond strength between nanoparticles and proteins and the progress of the equilibrium, Casals et al. [74] incubated citrate-silver nanoparticles (20 nm) in complete cell culture medium (cCCM), which consisted of Dulbecco’s modified Eagle’s medium [DMEM-10 % fetal bovine serum (FBS)] at a 10 % (v/v) dilution at 37 °C. These electrostatically stabilized silver nanoparticles were unstable when dispersed in solvents with a high ionic strength (e.g., DMEM) but stable when dispersed in the same solvents (e.g., DMEM) with 10 % FBS. Then, the adsorption/conjugation of proteins on the silver nanoparticle surfaces initiated as soon as the nanoparticles were dispersed in cCCM, which caused steric repulsion to occur more quickly than aggregation. In fact, the silver nanoparticle diameters were increased by up to twice the initial diameter. A simple purification process (centrifugation and resuspension in water) was carried out to distinguish the adsorbed protein from the free protein in solution. Part of the initial surface charge of the silver nanoparticles incubated with cCCM was recovered, indicating that only certain proteins remained attached to their surfaces and reached a stable/irreversible value. All data were indicative that the proteins had undergone a stabilization process, making them strong enough to maintain their final form as a hard protein corona over a long period of time in protein-free solutions. The authors suggested two steps in this process: (1) conjugation of a transient protein corona that forms immediately when nanoparticles contact the cCCM, which evolves a stable protein corona that remains bound to the nanoparticle surface even when the conjugates are taken out of cCCM and (2) redispersion in pure water (Scheme 1) [74].

**Fig. 6:**
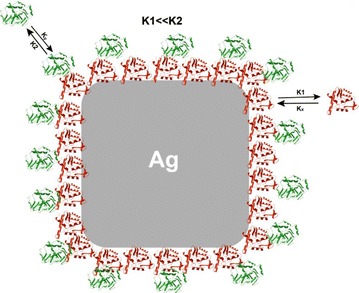
Schematic representation of a silver nanocubes surrounded by both slowly exchanging and rapidly exchanging proteins in a two-layer model (modified from ref. Miclaus et al. [40] by permission of ACS Publications)

Miclaus et al. [42] studied the interaction of silver nanocubes (synthesized by polyol synthesis and stabilized by PVP) with FBS, confirming the occurrence of a dynamic equilibrium process in which soft and hard coronas presented different exchange rates for this structure as well (Fig. 6).

**Fig. 7:**
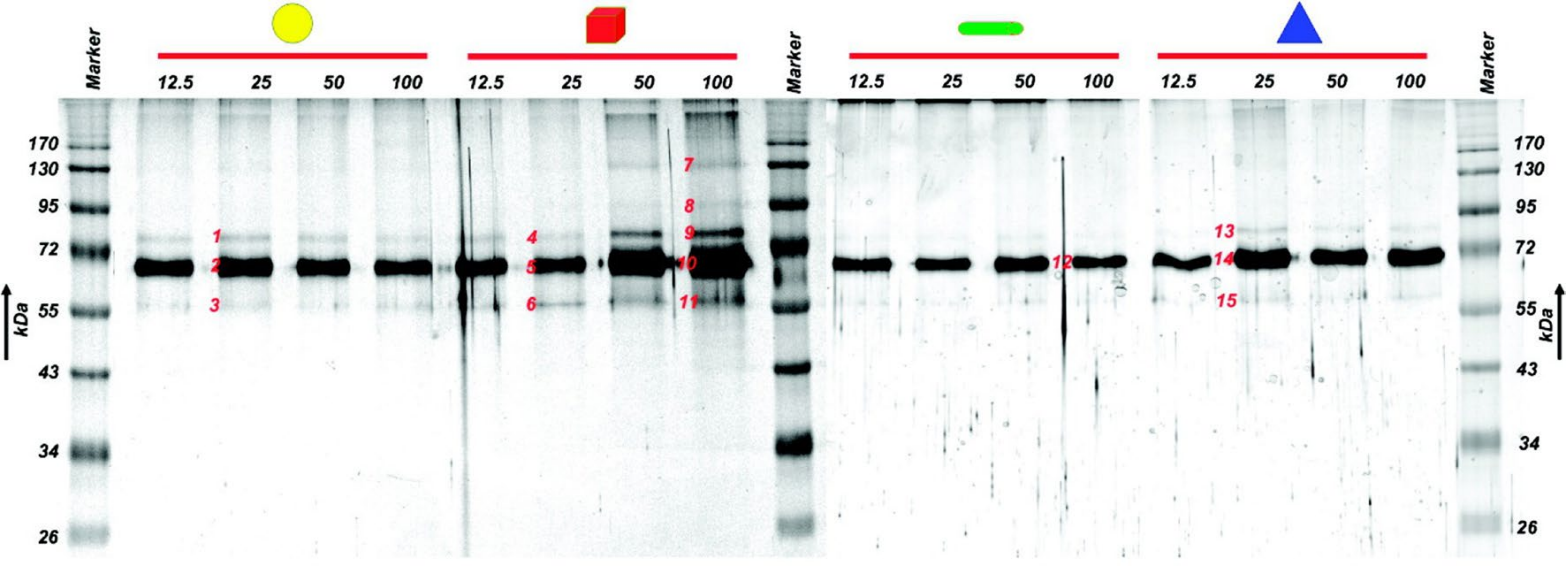
SDS–PAGE analysis demonstrate results in which various shapes and concentrations of silver nanoparticles were incubated in FBS, illustrating a clear difference in the protein corona composition and evolution trend for different shapes of particles (reproduced from ref. Ashkarran, et al. [1] by permission of American Chemical Society)

Ashkarran et al. [1] studied soft and hard corona effects, investigating the interactions of four different silver nanoparticle structures (cube, sphere, wire and triangle) with FBS in situ. SDS-PAGE analysis was conducted to demonstrate the interaction of the nanoparticles with the proteins in this fluid. The authors found that the protein coronas had different concentrations and compositions depending on the nanoparticle structure, indicating that protein-nanoparticle interactions are morphology-dependent (Fig. 7). Its is worth to mention that not only the curvature effects differ from one nanoparticle shape to another, but also the coordination of the atoms on the available surfaces, leading to different surface energies for each different morphology, which might play a role in the protein binding. A future full understanding of the morphology role on nanoparticle-protein interactions and a very detailed characterization of the corona are essential to the achievement of the desired effects of the nanoparticles in vivo, opening room to future studies that might link the right morphology according to the required effect.

Shannahan et al. [70] characterized citrate-silver nanoparticles (24.4 nm, −46.6 mV zeta potential) and PVP-silver nanoparticles (26.6 nm, −35.9 mV zeta potential) in water or DMEM cell culture medium. A slight effect was observed when silver nanoparticles were suspended in DMEM without serum. From the results, it was clear that citrate-stabilized silver nanoparticles in water exhibited more negative zeta potentials than did PVP-stabilized nanoparticles of a similar diameter. Analyses of the composition of the protein coronas for a set of four silver nanoparticles, including citrate-stabilized and PVP-stabilized colloidal silver (~20 or ~110 nm diameter), were accomplished. The silver nanoparticles were incubated in DMEM supplemented with 10 % FBS, then washed several times, and the protein corona was solubilized and analyzed by label-free LC–MS/MS. The silver nanoparticles were found to be associated with a common subset of 11 proteins, including albumin, apolipoproteins, keratins, and other serum proteins. In fact, for the 110 nm citrate- and PVP-stabilized silver nanoparticles, the results showed binding to 79 and 85 proteins, respectively, and for the 20 nm citrate- and PVP-stabilized nanoparticles, the results showed binding of 45 and 48 proteins, respectively. The authors suggest that this is an indication that surface curvature effects are ruling the protein corona formation. Regarding the specificity of the silver nanoparticles studied, 19 proteins bound only the 20 nm silver nanoparticles (PVP and citrate), whereas 60 bound only the 110 nm silver nanoparticles (PVP and citrate). The authors indicate that these different protein profiles may also give a protein corona signature that is based on the size and/or surface curvature of the silver nanoparticles. However, it is important to point out that the total surface area available related to each of the nanoparticles studied (~20 and ~110 nm diameter) are completely different, which might affect the amount and composition of proteins binding to the nanoparticles. The difference in the total surface area available to bind proteins results from normalizing by weight the amount of nanoparticles used in the corona studies, in which the authors incubated 1 mg of each nanoparticle with the media. This highlights the need to start considering the total surface area as a parameter to be controlled in this type of studies.

#### Human blood plasma

Silver nanoparticles may supply antimicrobial efficacy when used as coatings on several biomedical devices. Therefore, it is very important to understand the interactions of silver nanoparticles with blood components (e.g., RBCs, WBCs, plasma proteins, etc.) [23, 75]. In human plasma, a typical nanoparticle protein corona consists of proteins such as HSA, immunoglobulins, fibrinogen, apolipoproteins, transferrin, complement proteins, and hemoglobin. The adsorption pattern of blood proteins to external inorganic surfaces is a functional event in which the most abundant proteins (e.g., HSA and fibrinogen) may initially be absorbed by the surface and then later replaced by other proteins that have a greater binding affinity for the surface (i.e., Vroman theory) [76]. However, there is no universal theory that can be applied to all types of nanoparticles when exposed to human plasma [77]. Human plasma protein corona formation on metallic nanoparticles (i.e., gold and iron) has been reported [78, 79]. However, we have not found reports in the literature for silver nanoparticles (to the best of our knowledge). Recently, using surface-enhanced Raman spectroscopy (SERS), interactions of silver nanoparticles with normal and cervical cancer human plasma collected from patients were reported [80]. In this specific case that action of proteins and silver nanoparticles were used as markers for differentiation of normal and cancer human plasma. The prominent SERS peak is attributed to the amide I band of the human serum albumin (HSA) and in cancer this band was found to be higher than normal plasma, indicating that the elevated relative amount of proteins in the α-helix conformation was due to the cancer development. In other words, the selective proteins in cancer exert a different interaction with silver nanoparticles than normal plasma. These findings point towards the importance and role of the medium in the protein-nanoparticle interactions. Future studies that focus on a complete and accurate characterization of the corona and subsequently in the comparison of its composition in different disease conditions might give valuable information about the key proteins involved in the development and progress of certain illnesses.

#### Human serum albumin (HSA)

A study by Gebauer et al. [81] showed that the protein corona formed by human serum albumin (HSA) on citrate-functionalized silver nanoparticles stabilized them against agglomeration. One important aspect of this research was that agglomeration kinetics can be employed to prescribe apparent affinities to describe the protein adsorption/desorption equilibrium. Furthermore, circular dichroism spectroscopy was used as a complementary technique to measure affinities for the same process. The apparent K_D_ values obtained using both approaches were correlated with other published values. The protein coronas found around silver nanoparticles in these experiments consisted of a monolayer of protein on the nanoparticle surfaces, corroborating previous results of HSA adsorption to polymer-coated nanoparticles [50].

Chen et al. [82] studied the interactions between citrate-silver nanoparticles and HSA using several methods, including UV–Vis, TEM, and CD measurements, and found that silver nanoparticle-HSA binding was mediated by the physical forces of hydrogen bonding, electrostatic interaction, and hydrophobic interaction. In this study, the percent of α-helices was reduced, whereas that of β-sheets increased in the secondary HSA structures after interaction with silver nanoparticles. The authors suggested that this was a result of hydrogen bond breakage between proximal α-helices and configuration of new, less ordered hydrogen bonds between the α-helices and the citrate coating of the silver nanoparticles. CD measurements indicated that the presence of lipid vesicles (i.e., the membrane model) relaxed the conformational changes of the proteins induced by the nanoparticles, most likely due to the electrostatic repulsion between them. Generalized polarization (GP; fluorescence ratio) measurements demonstrated that nanoparticles and protein coronas interact with lipid vesicles to enhance their fluidity. In contrast, free proteins did not exert this effect on vesicle conformation. Utilizing these results, the authors suggested that in the formation of nanoparticle protein coronas, the physical interactions between the nanoparticle core and cell membranes probably did not occur easily. The implications of these results together with the biological and biochemical mechanisms of endocytosis, lipid peroxidation, and enzymatic activity, could be an important issue in nanomedicine and nanotoxicology applications [82].

After a 2-h pre-incubation with proteins (HSA/transferrin), citrate-silver nanoparticles (20 and 110 nm) were studied as they acted on human epidermal keratinocyte (HEK) cells, and the cellular uptake showed that for 20 and 110 nm citrate silver nanoparticles, protein incubation significantly modulated silver nanoparticle uptake. Silver incubation with albumin and IgG, and in a minor extension with transferrin, reduced the extent of cellular uptake [81]. Moreover, IgG exposure dramatically reduced the 110 nm citrate silver uptake. Similar profiles were also found with silver nanoparticles in the membrane fraction with protein incubation in general, significantly altering the association of the particle to the membrane. Silver nanoparticles, independent of size and protein exposure, were found inside the cytoplasmic vacuoles of the HEKs.

**Fig. 8:**
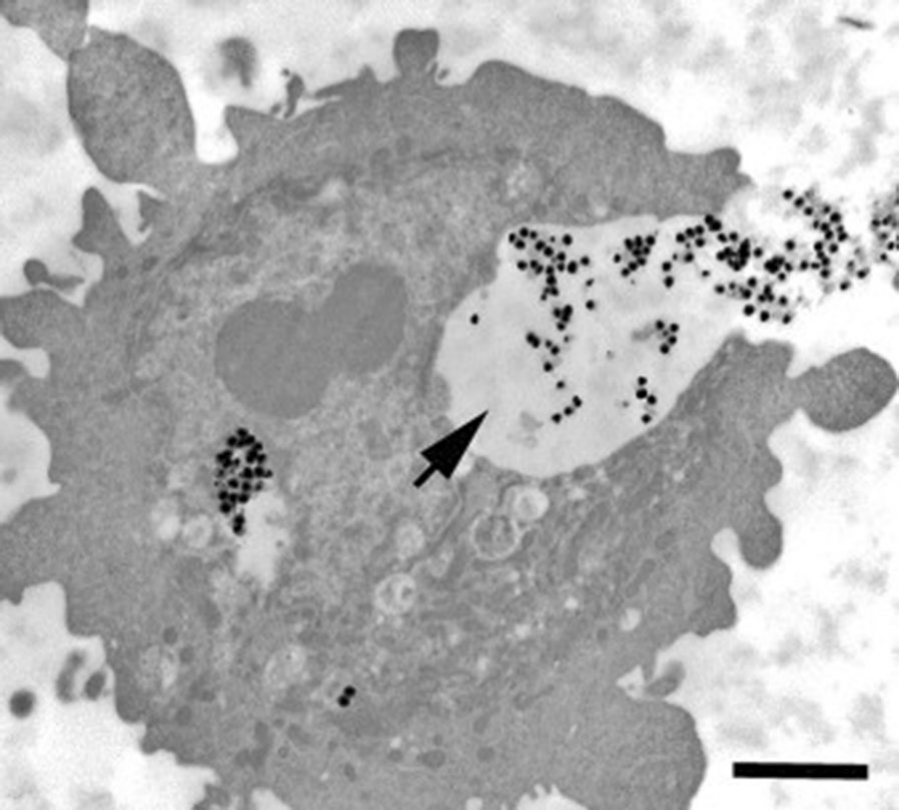
Transmission electron micrograph of an HEK engulfing 120 nm silica-coated silver nanoparticles. *Arrow* denotes silver nanoparticles within invagination. *Bar* 2 µm (reproduced from ref. Monteiro-Riviere et al. [81] by permission of Elsevier Ireland Ltd.)

Figure 8 shows a transmission electron micrograph that has captured several 120 nm silica-coated silver nanoparticles in the process of phagocytosis by a HEK cell.

The 20 nm citrate silver nanoparticles bound to albumin were highly dispersed and appeared to break down or disintegrate within the vacuoles, whereas nanoparticles bound to IgG, transferrin, and the native control showed very low agglomeration. However, for 20 nm citrate coated silver nanoparticles, protein incubation reduced cellular concentrations, and for the 110 nm citrate silver nanoparticles, incubation with IgG significantly reduced the uptake of silver nanoparticles. The authors stated that this pattern of protein modulation of silver nanoparticle uptake was not as important compared with that of different proteins that clearly modify silver nanoparticle uptake into HEK. They also discussed the cytoplasmic data that showed that nanoparticles certainly entered the cellular domain, which brought up questions as to how in vitro assays using native nanoparticles could be correlated to what would be expected to occur in vivo [83].

In the case of HAS, the incubation significantly modulated silver nanoparticle uptake independently of the nanoparticles sizes. The incubation with albumin and IgG, and in a minor extension with transferrin, reduced the extent of cellular uptake.

#### Tubulin (cytoskeletal protein)

**Fig. 9:**
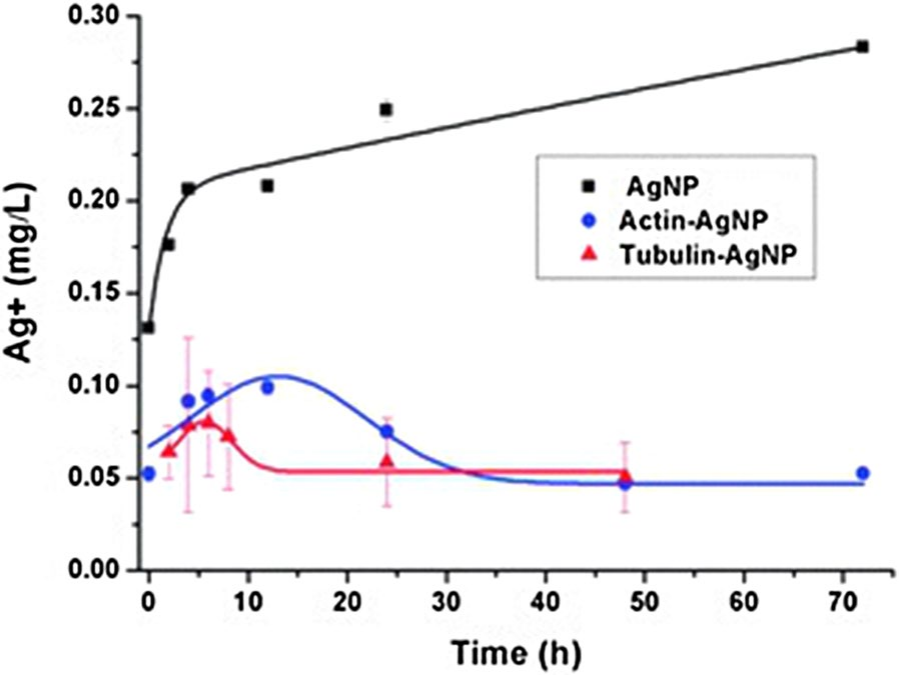
Release of silver ions with and without the presence of proteins, measured (n = 3) by ICP–MS. Original silver nanoparticles (AgNP) concentration: 5 mg L^−1^. Actin and tubulin concentrations: 5 mg L^−1^ (reproduced from ref. Wen et al. [82] by permission of The Royal Society of Chemistry)

In a study with actin and tubulin (cytoskeletal proteins) interacting with citrate-silver nanoparticles, both gave high standard deviations for the zeta potentials. The silver nanoparticles had a diameter of 35.7 nm and a zeta potential of −42.5 mV and actin had a diameter of 2.0 nm and a zeta potential of −28.0 mV, while actin-silver nanoparticles had a diameter of 39.4 and a zeta potential of −36.6 mV. It was suggested that this was due to self-aggregation and less extensive polymerization. The actin-silver nanoparticles exhibited a smaller deviation in zeta potential than the tubulin-silver nanoparticles, indicating that the protein corona from actin-silver nanoparticles was more homogeneous than the tubulin-silver nanoparticles. As discussed previously, the secondary structures of actin and tubulin were altered after interaction with silver nanoparticles. The α-helices of actin showed a 24 % decrease whereas the β-sheets showed a 36 % increase upon binding to the silver nanoparticles, and no random coils were observed. For tubulin, a 17 % decrease and 5 % increase in α-helices and β-sheets, respectively, was demonstrated. In the latter protein, 11 % random coils were found after binding with silver nanoparticles. The conformational changes were more significant for actin than tubulin, corroborating the UV–Vis absorbance measurements and the hyperspectral imaging. Other important aspects of this report included silver ion release in these types of interactions. In the absence of cytoskeletal proteins, silver nanoparticles rapidly released silver ions for the first 4 h, but the rate of release subsequently leveled off for a period of 72 h (5 % dissolution). In the presence of actin and tubulin, however, the release of silver ions progressed at a slower pace, where only 1 % dissolution was attained during the first few hours (Fig. 9).

These data imply that coating silver nanoparticles with cytoskeletal proteins physically prevented release of the silver ions, and the conformation and physicochemical properties of silver nanoparticles were improved by hardened cytoskeletal proteins [84].

#### Ubiquitin (a structurally conserved protein that regulates numerous in eukaryotic cell processes)

**Fig. 10:**
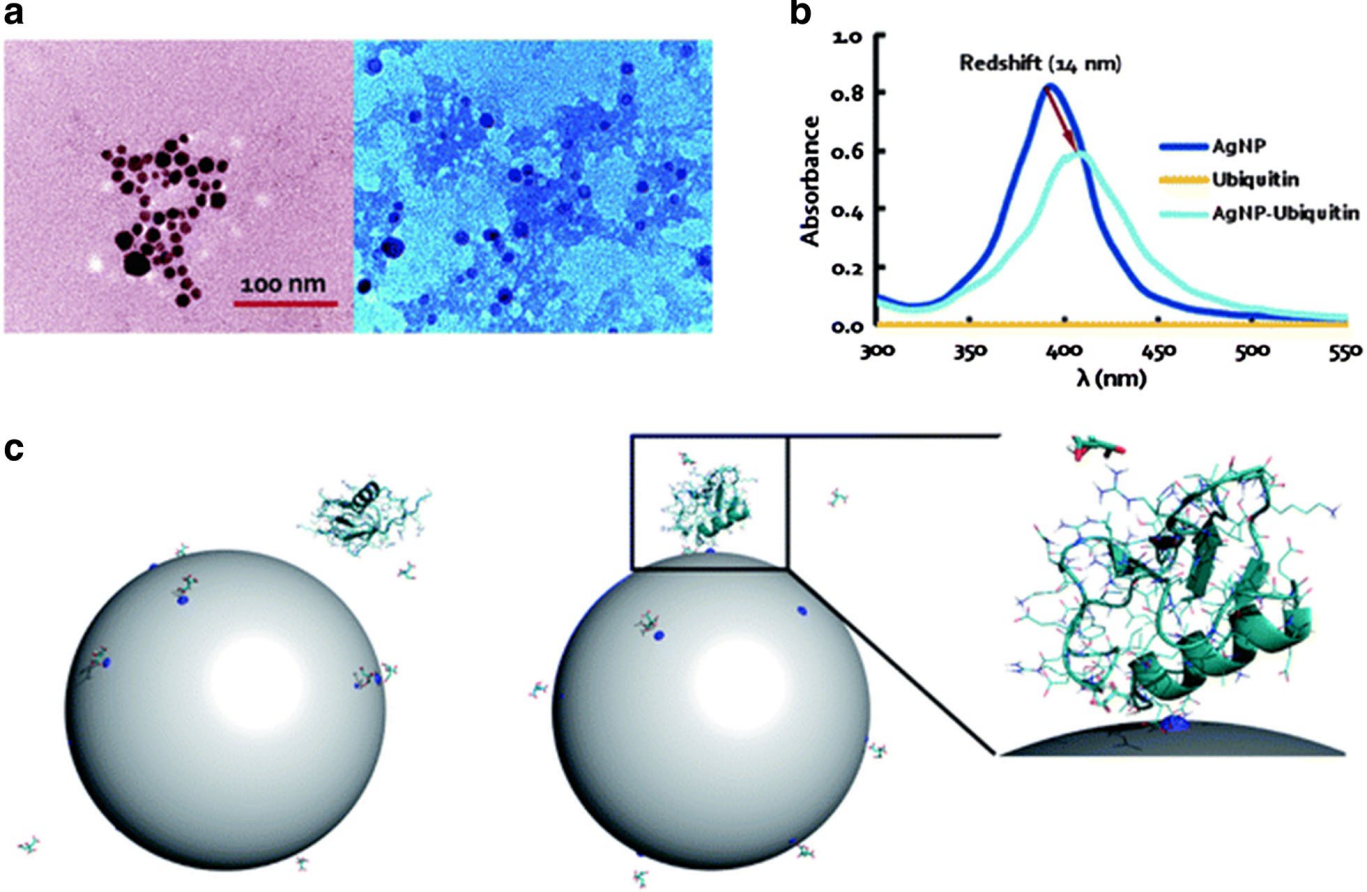
Interaction between a single ubiquitin and a citrate-coated silver nanoparticles. **a** TEM images of silver nanoparticles (*left panel*) and silver nanoparticles-ubiquitin coronas (*right panel*) where the associations of the silver nanoparticles and the proteins (*shaded regions*) are evident to imply their good binding affinity. **b** UV–vis absorbance of silver nanoparticles, ubiquitin, and silver nanoparticles–ubiquitin, featuring a red-shift of the absorbance peaks for silver nanoparticles–ubiquitin and silver nanoparticles alone due to dampened surface plasmon resonance. **c** Initial (t = 0 ns) and final (t = 50 ns) structure of the ubiquitin–citrate–silver nanoparticles complex system. Ubiquitin is represented as *cartoons*, the side chains as *lines*, and the citrates as *sticks*. The *gray sphere* represents the nanoparticle, and the charged atoms on the silver nanoparticles surface are shown as *blue spheres*. The zoom-in view of the final structure indicates the binding between the ubiquitin and a charged silver nanoparticles surface atom (modified from Ding et al. [83] by permission of The Royal Society of Chemistry)

Ding et al. [85] used a combination of molecular dynamics simulations and complementary experiments to characterize the ubiquitin corona on citrate silver nanoparticles of around 13 nm (Fig. 10). To measure the impact of silver nanoparticles on the ubiquitin conformation, the Q-value was studied for all protein residues, which is the fraction of native contacts for silver nanoparticles when bound to ubiquitin or unbound. The Q-values were close to 1, indicating that both components maintained native-like structures. The loop regions between the secondary structures had relatively low Q-values. A region near the C-terminus of the helix and another close to residue 46 in a loop were strongly destabilized upon binding with silver nanoparticles. These data were consistent with results from circular dichroism, where the helical content was reduced by 27.8 % by the silver nanoparticle-bound ubiquitin compared to free ubiquitin. These measurements also showed a slight increase in the β-sheet content. The authors hypothesized that as a function of protein concentration on the silver nanoparticle surfaces, the increase in β-sheet content could be due to the formation of inter-protein hydrogen bonds through partially unfolded protein regions.

#### Hyaluronan-binding protein

Hyaluronan is a major component of cell-surface glycoproteins and the extracellular matrix, surface adsorbed hyaluronan-binding proteins may act as “bridges” that mediate the interaction of nanoparticles with the cell surface. Recently Walkey et al. [86] noted that using quantitative models (a bioinformatics-inspired approach) to predict the biological interactions of nanoparticles that they included for the characterization of the blood protein coronas is the most comprehensive quantitative analysis published to date. The model implicated a collection of hyaluronan-binding proteins as mediators of silver nanoparticle-cell interactions. They also indicated the probability of using this database to develop exact relationships that can predict the biological responses to nanoparticles and potentially help in uncovering the basic nano-bio interaction mechanisms.

#### Yeast extracted proteins

**Fig. 11:**
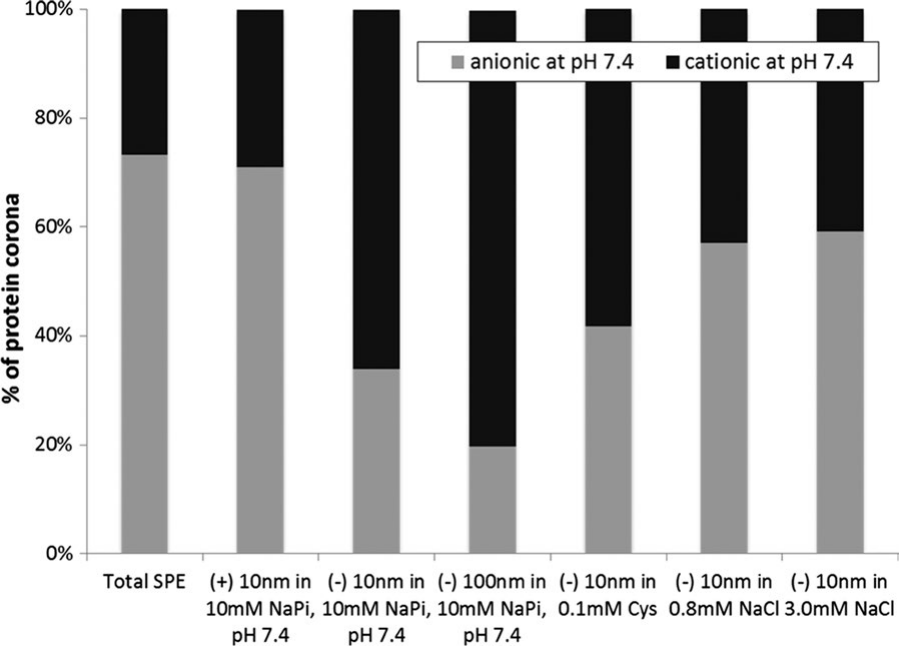
Percent abundance of protein pIs across the six sample conditions tested (reproduced from ref. Eigenheer et al. [85] by permission of The Royal Society of Chemistry)

Eigenheer et al. [87] reported an approach where mass spectrometry proteomics was implemented to determine protein corona populations (from yeast extract) and to measure protein enrichment on the surfaces of different silver nanoparticle [negative citrate-coated particles (10 and 100 nm) and positive branched polyethyleneimine coated particles (100 nm)] under biologically and environmentally important reaction conditions. BY4 yeast was cultured and the cells treated to obtain the protein content. After incubation of 0.25 mg mL^−1^ nanoparticles and 0.21 mg mL^−1^ soluble yeast protein extract for 16 h, the nanoparticles and adsorbed proteins were precipitated, centrifuged and washed several times to remove non-associated and loosely associated proteins from the nanoparticles. Nanoparticle adsorbed proteins were directly digested off the nanoparticles (e.g., trypsin) for identification of well adsorbed and low abundance corona proteins. More than 500 proteins were identified in the sample studied, including nanoparticles with associated and unassociated proteins. To understand the role of silver nanoparticle properties in protein corona formation, the protein–nanoparticle interaction was carried out with low salt and buffer concentrations. The protein coronas of citrate-silver nanoparticles (10 nm, negative) were compared to polyethyleneimine-silver nanoparticles (10 nm, positive) and citrate-silver nanoparticles (100 nm, negative) to investigate the roles of surface charge and size, respectively. The data from the protein corona concentration experiments clearly indicated that the charge on the surface of the silver nanoparticles played a greater role in corona formation than did their size. The authors presented a Venn diagram to compare the different protein corona populations and found that 10 and 100 nm (negative) citrate-silver nanoparticles were the most similar, with approximately 83 % of proteins (376 proteins) shared across their protein corona distributions. Meanwhile, the differences provided insight into the importance of nanoparticle properties. Notably, approximately 50 % of the proteins (247 proteins) were found within the coronas of all nanoparticles studied, which demonstrated that these proteins would be found on the nanoparticles independent of their surface coating or size and are therefore an important group of proteins that must be studied in further detail. The authors also suggested that the surface charge, an apparent biophysical property of the proteins studied, probably regulates binding to silver nanoparticles, as shown clearly in Fig. 11. Here, there was an increase in the abundance of negatively charged proteins that bind to (+) silver nanoparticles compared to the 10 and 100 nm (−) silver nanoparticles. In other words, there was a two-fold decrease in the anionic proteins bound to 10 nm (34 %) and 100 nm (−) silver nanoparticles (20 %), compared to (+) silver nanoparticles (71 %). These data demonstrated that electrostatics is a determinant factor in the modulation of protein affinity for silver nanoparticles. Finally, the authors stated that, at the most basic level, all of this information reaffirms the importance of ample nanoparticle characterization and the reaction conditions when analyzing the protein coronas of nanoparticles.

### Biological activities related to the protein corona

#### Antibacterial activity

The antibacterial activities of borate-silver, citrate-silver and PVP-silver nanoparticles were studied. Borate-silver nanoparticles were inactive against *Salmonella* when tested in Luria–Bertani (LB) broth, but were efficient against *Salmonella* in PBS buffer. When antibacterial activity was tested in PBS (no blood, serum or plasma present), no activity differences were found for the different types of silver nanoparticles. However, blood, serum or plasma completely inhibited the activity of borate-silver nanoparticles but not citrate-silver or PVP-silver nanoparticles. The activity of the PVP-silver nanoparticles was stronger than that of the citrate-silver nanoparticles. Nanoparticle activity against *S. typhimurium* was studied in the presence of mouse blood components, whereas for *S. typhi*, the activity was analyzed in the presence of human blood components. Serum albumin is the major component of blood; 3 % BSA was used to measure the antibacterial effects of the silver nanoparticles under study. BSA addition totally abolished the antibacterial activity of the borate-silver nanoparticles but not that of the citrate- or PVP-silver nanoparticles [88].

Citrate- and PVP-silver nanoparticles presented antibacterial properties in LB broth. The authors suggested that this might be due to the prevention of interaction of silver nanoparticles via the LB components or the inhibition of agglomeration of these particles.

A murine salmonellosis model was used by the same authors to understand the effect of the capping agent on the silver nanoparticles. BALB/c mice were infected with *S. typhimurium* (orally/i.v.), and the bacterial burdens in the mesenteric lymph nodes, spleen, and liver were studied. No effect of silver nanoparticles was observed when borate-silver nanoparticles (described as uncapped-silver nanoparticles) were administered to treat the *Salmonella* infection, whereas citrate- and PVP-silver nanoparticles reduced the bacterial burden in different organs; PVP-silver nanoparticles showed an efficient antibacterial effect. Finally, when the borate-silver nanoparticles were used, all mice died after the same period as in the controls, whereas oral and i.v. administration of citrate- or PVP-silver nanoparticles delayed the death of the mice. However, when the silver nanoparticles were all administered intravenously, the PVP- and citrate-silver nanoparticles protected 60 and 40 % of the mice from death, respectively. This difference between oral and i.v. administration was attributed to the fact that silver nanoparticles from oral administration were excreted through the feces. The authors noted that this study was the first to show the role of the capping agent in antibacterial activity under physiological conditions in an animal model and also how the efficacy of silver nanoparticles varies between PBS and biological fluids [88]. As previously discussed, not only in in vitro studies but also in vivo antibacterial activity of silver nanoparticles depends on the capping agents.

#### Cytotoxicity

Citrate-silver nanoparticles were found by Shannahan et al. [41] to readily associate and form protein coronas with human serum albumin (HSA), bovine serum albumin (BSA), and high density lipoprotein (HDL). The accumulation of all protein coronas increased the hydrodynamic sizes of the silver nanoparticles. The addition of HSA and BSA reduced the dissolution of silver nanoparticles (silver ion formation), whereas, for HDL, dissolution increased compared with silver nanoparticles in water. The authors suggested that this occurred because of a reduction in the zeta potential of the HDL-silver nanoparticles compared with other samples. Moreover, HDL probably offset citrate groups and produced a destabilization of the silver nanoparticles, generating an increase in total dissolution. Circular dichroism measurements showed that protein association with silver nanoparticles decreased the number of α-helices in all protein coronas, but for HDL-silver nanoparticles, the number of α-helices increased. For HSA and BSA, this increase probably arose from entropy-driven unfolding of the protein and the formation of hydrogen bonding between the proteins and the silver nanoparticles. However, for HDL, this probably did not occur because this material has a high lipid content and a high degree of structural complexity. Additionally, the number of β-sheets increased in all proteins, and protein turns as well as the quantity of unordered proteins were also discussed. An important topic investigated by this research was the role of the protein corona in the toxicity induced by silver nanoparticles in two cell types that are common targets of nanoparticle exposure, rat lung epithelial (RLE) and rat aortic endothelial (RAEC) cells. As observed by hyperspectral analysis, the protein coronas on the silver nanoparticles were lost after internalization and these nanoparticles activated cells by inducing IL-6 mRNA expression. Pretreatment with an SR-BI inhibitor (inhibitor of the HDL receptor) diminished the internalization of silver nanoparticles both with and without a protein corona, and it reduced cytotoxicity and IL-6 mRNA expression. Therefore, this study characterized the formation of a protein corona on silver nanoparticles and showed its influence on cell activation and cytotoxicity through cell surface receptors.

**Fig. 12:**
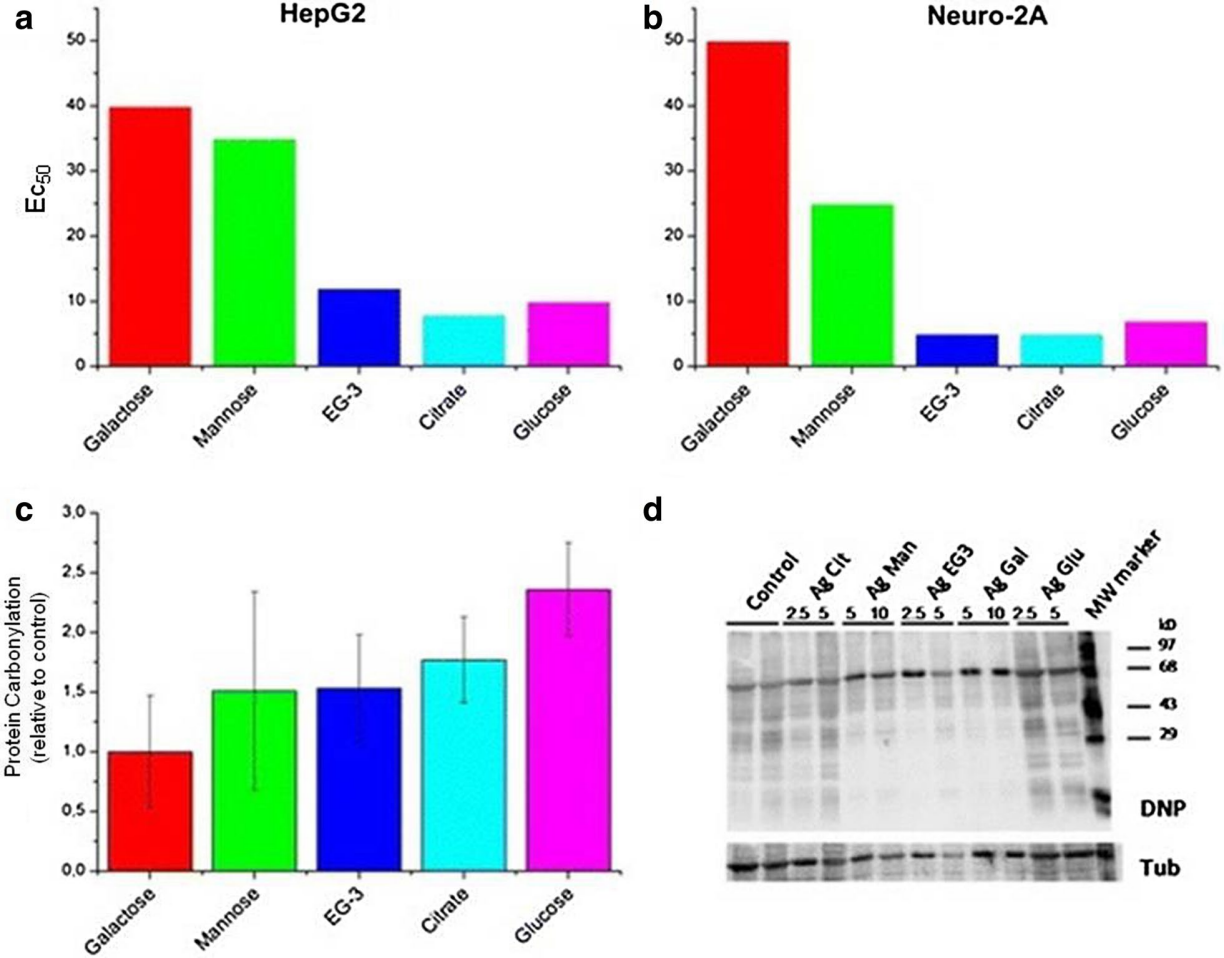
Toxicity results in vitro. **a** EC50 values from MTT assay using silver nanoparticles with different coatings and HepG2 cells; **b** analogous with Neuro-2 cells; **c** detection of oxidative stress from silver nanoparticles (concentration 5 pM) via formation of protein carbonyls incubated with HepG2 cells. **d** Protein carbonyls were detected at different concentrations (2.5, 5, 10 pM) as (DNP) hydrazone adducts via immunoblots with a DNP antibody (reproduced from ref. Kennedy et al. [87] by permission of BioMed Central)

In 2014, citrate-silver nanoparticles functionalized with three different thioglycosides (glucose-, galactose-, and mannose-silver nanoparticles) and thioglycol-silver nanoparticles were synthesized by Kennedy et al. [89]. The cytotoxicity of these functionalized silver nanoparticles were tested against a neuron-like cell line (Neuro-2A) and a hepatocyte cell line (HepG2). The coating was clearly important for their toxicity. Glycol-, glucose- and citrate-functionalized silver nanoparticles exhibited a similarly high toxicity. However, galactose- and mannose-functionalized nanoparticles were significantly less toxic towards both cell lines. The formation of protein carbonyls was analyzed as an indirect mark for the oxidative stress induced by nanoparticles because proteins can become carboxylated either as a direct or an indirect consequence of reactive oxygen species formation (Fig. 12).

The authors found a strong correlation between carbonyl formation and EC50 values, suggesting that toxicity is predominantly caused by the oxidative stress related to ROS formation. However, it is also possible that toxicity can arise from different cellular uptake rates. An ICP-MS and confocal microscopy experiment showed that for both cell lines, the less toxic galactose-functionalized nanoparticles were taken up more efficiently compared to mannose- or glucose-functionalized particles, which was probably due to a galactose-specific receptor on the surface of the cells. In other words, higher cell internalization does not necessary indicate higher toxicity. The glucose-silver nanoparticles caused the highest toxicity and protein carboxylation but not the highest cellular uptake, when compared with the other functionalized nanoparticles. A confocal microscopy image of the Neuro-2A cells showed that the galactose-silver nanoparticles were mainly inside the cytoplasm (e.g., inside vesicular structures) but not in the nucleus. For the mannose- and glucose-silver nanoparticles, their clusters appeared more evenly dispersed throughout the cell, and the intracellular clusters were smaller than those of particles with other functional groups. Another aspect that these authors discussed was the importance of surface charge for cellular internalization. In these experiments, there was a correlation between the uptake and surface charge for mannose, glucose and ethylene glycol silver nanoparticles, which are more negatively charged and taken up less efficiently. These data were consistent with their expectations because the cell lines used were also negatively charged owing to their various carbohydrate moieties. However, the authors also suggested that in addition to special receptors on the cell surface, there are also protein coronas on their surfaces from interaction with the culture medium. Zeta potential measurements after incubation showed that all particles exhibited similar overall negative charges, confirming the formation of protein coronas. Presumably, the protein corona composition on the surface of the functionalized particles in this study was expected to be different depending on particle coating, and the authors clearly found a correlation between particle coating, oxidative stress and toxicity [89]. It is clear from this study that the formation of a protein corona on silver nanoparticles influenced cell activation and cytotoxicity through cell surface receptors.

## Conclusions

Based on all data related to silver nanoparticles and their protein coronas and depending on factors such as size, concentration and surface functionalization, unexpected interactions with different blood components or any biological fluids could interfere with biomedical applications, such as antibacterial, antifungal or anticancer treatment in vivo. The scientific community is still in the beginning of the exploration of this new subject and interestingly, all studies conducted to this end have focused on human plasma as the test system for protein corona studies. Though of extremely importance to mimic and study biological interactions with these nanomaterials, the use of this model is still deficient for studies with silver nanoparticles. We could see from this review that circular dichroism was extensively used to confirm the interactions between single proteins and silver nanoparticles, confirming a dynamic type of interaction that could lead to protein unfolding. Extending these findings to a biological milieu, the changes in the secondary structure of proteins as a result from protein–nanoparticle interaction could lead to different cell responses and activation processes, which is still in need of further investigation. Understanding the interaction of these protein coronas with cells could lead to understanding of their immunotoxicity or its reduction by silver nanoparticles capped with protein corona. In some cases, silver nanoparticle cytotoxicity was mitigated, depending on protein corona composition. Recently Lee et al. [43] stated that there is limited understanding of the connection between the physicochemical properties of any nanoparticle and their corresponding effects on physiological systems. We agree with this statement in the context of silver nanoparticles. However, depending of the application route, many advances for in vivo applications have been made by functionalization. We will probably see in vivo applications of silver nanoparticles in the near future. However, a major challenge will be to compare data across interlaboratory studies, which is found to be difficult so far as most of them do not follow same conditions. This reveals an urgent need to better control the parameters of the experiments as well as to standardize some of conditions, e.g. particle concentration, total surface area available and biological fluids conditions, thus making possible to build a strong and solid database.
